# Distribution of HIV-1 subtypes and molecular transmission networks among HIV/AIDS cases in Dinghai District, Zhoushan, China

**DOI:** 10.1097/MD.0000000000050649

**Published:** 2026-09-11

**Authors:** Youshu Wang, Bo Zhang, Songye Gu, Qizhen Yu, Junwei Chen

**Affiliations:** a HIV/AIDS and Tuberculosis Department, Dinghai Center for Disease Control and Prevention, Zhoushan, Zhejiang, China; b Clinical Laboratory, Zhoushan Center for Disease Control and Prevention, Zhoushan, Zhejiang, China; c Clinical Laboratory, Dinghai Center for Disease Control and Prevention, Zhoushan, Zhejiang, China.

**Keywords:** circulating recombinant forms, clustering analysis, epidemiology, HIV-1, molecular transmission network, subtypes, unique recombinant forms

## Abstract

This study examined the molecular transmission characteristics of human immunodeficiency virus type 1 (HIV-1) among newly diagnosed HIV/acquired immunodeficiency syndrome cases in Dinghai District, Zhoushan City, Zhejiang Province, with the aim of informing targeted prevention and control strategies. Newly reported HIV/acquired immunodeficiency syndrome cases from 2022 to 2024 were enrolled, and demographic as well as epidemiological information was collected. Plasma samples obtained prior to antiretroviral therapy were subjected to reverse transcription and nested polymerase chain reaction to amplify the polymerase gene. HIV-1 subtypes were determined using Neighbor-Joining phylogenetic analysis, and molecular transmission networks were constructed with Cytoscape 3.6.1 based on Tamura-Nei 1993 (model) genetic-distance thresholds. A total of 147 cases were identified during the study period, among which 121 blood samples were collected and 117 high-quality polymerase gene sequences were successfully obtained. Of these cases, 87.18% were male, 46.15% were aged ≥50 years, and 71.79% had a junior high school education or below. Commercial heterosexual contact was the most frequently reported route of transmission (38.46%). Thirteen HIV-1 subtypes were identified with CRF07_BC (37.61%) and CRF01_AE (23.93%), being the predominant strains. Using a genetic-distance threshold of 1%, 14 molecular clusters comprising 36 sequences were identified, corresponding to an overall clustering rate of 30.77%. The largest cluster included 10 individuals. Notably, 9 high-risk individuals with 4 or more network links were all infected with CRF07_BC and were predominantly older males with lower educational levels who reported commercial heterosexual exposure. In conclusion, CRF07_BC and CRF01_AE were the dominant HIV-1 subtypes circulating in Dinghai District. Middle-aged and older individuals involved in commercial heterosexual activities constituted the core of the local transmission network. Strengthening targeted interventions and expanding HIV testing coverage in this population are essential to prevent further transmission. These findings indicate that molecular transmission-network analysis can provide useful district-level evidence for identifying potential priority populations and optimizing targeted HIV prevention strategies in Dinghai District. The group with higher clustering signals may play an important role in local transmission, but molecular links should not be interpreted as proof of direct transmission.

## 1. Introduction

Human immunodeficiency virus (HIV) remains a major public health concern. In 2024, an estimated 1.3 million people acquired HIV and 630,000 died of HIV-related causes worldwide, underscoring the need for prevention strategies that are informed by local transmission dynamics. Molecular epidemiology, linking viral sequence diversity with inferred person-to-person connectivity, has become central to identifying rapidly growing clusters and prioritizing interventions. HIV-1 diversity shapes epidemic trajectories. In China, long-term national surveillance indicates that circulating recombinant forms (CRFs) CRF07_BC and CRF01_AE have dominated recent epidemics, with CRF08_BC, subtype B, and CRF55_01B contributing smaller fractions. Analysis of polymerase (pol) gene sequences from 2004 to 2023 estimated proportions of 39.1% for CRF07_BC, 32.1% for CRF01_AE, and 9.2% for CRF08_BC among newly reported infections, reflecting sustained recombination and intermixing across geographies and risk groups.^[1]^ In parallel, regional investigations continue to identify novel CRF01_AE/CRF07_BC recombinants and unique recombinant forms (URFs), reinforcing the need for ongoing genomic surveillance.^[2]^

Eastern China, including Zhejiang Province, exhibits pronounced heterogeneity in subtype composition and network features. Recent work in Zhejiang characterized major subtypes and reconstructed transmission networks, highlighting complex recombination patterns and geographically structured clusters that can inform focused public health responses.^[3]^ Importantly, several contemporary studies from eastern and central China show that older adults (≥50 years) increasingly contribute to transmission networks and may harbor high node degree or elevated pretreatment drug resistance, pointing to under-recognized prevention gaps in this demographic.^[4]^ Concurrently, men who have sex with men (MSM)-centered networks remain epidemiologically important in many settings; molecular-network analyses have documented large CRF07_BC-dominant clusters among MSM and potential bridging to heterosexual networks through individuals with dual partnerships, emphasizing the need to monitor cross-network spillover.^[5]^ Molecular transmission-network analysis typically uses pol sequences, the Tamura-Nei 1993 (TN93) model genetic-distance model, and empirically selected thresholds to infer putative linkages. When triangulated with demographic and behavioral data, these networks can reveal cluster growth over time, identify high-degree cases that may sustain onward spread, and localize micro-environments where venue-based testing and partner services could be impactful. In China, methodological standardization and the scaling of near–real-time analytics have expanded the utility of network-informed interventions in routine surveillance.^[6]^

Dinghai District of Zhoushan City is a coastal area characterized by commercial activity, tourism, and population mobility – conditions that can facilitate repeated introductions and local amplification. Yet district-level evidence linking subtype composition to network structure remains sparse. Building on the emergent literature from Zhejiang and other provinces, there is a need to define local subtype landscapes, quantify clustering, and assess whether specific demographic strata – particularly older adults and populations involved in commercial heterosexual contact – disproportionately anchor recent transmission.^[3]^ Accordingly, we conducted a molecular epidemiology study of newly diagnosed HIV/acquired immunodeficiency syndrome (AIDS) cases in Dinghai District (2022–2024) to characterize the HIV-1 subtype/CRF/URF spectrum based on pol sequences; construct molecular transmission networks using TN93 distances at a prespecified threshold and delineate cluster architecture; and examine demographic and epidemiologic correlates of clustering, with attention to age, marital status, education, residence, and reported exposure category. By aligning subtype diversity with network topology, the study aims to generate actionable evidence for targeted screening, partner notification services, venue-focused outreach, and rapid treatment initiation within the district.

## 2. Methods

### 2.1. Study population

All newly diagnosed HIV/AIDS cases reported in Dinghai District, Zhoushan City, Zhejiang Province, between January 2022 and December 2024 were considered for inclusion. Eligible participants were individuals who were confirmed HIV-1 positive at the time of their first follow-up and who provided informed consent. Demographic and epidemiological data, including sex, age, place of residence, occupation, marital status, educational attainment, and reported route of HIV acquisition, were collected through standardized questionnaires and medical records. The study protocol was reviewed and approved by the Ethics Committee of the Dinghai District Center for Disease Control and Prevention (approval number: DHJK-2023-004).

### 2.2. Sample collection and nucleic acid extraction

At baseline, 6 mL of peripheral venous blood was collected into anticoagulant tubes. Plasma was separated by centrifugation at 3000 rpm for 10 minutes and stored at −80°C until further analysis. Viral ribonucleic acid was extracted from plasma using a commercial nucleic acid extraction kit (Promega Corporation, Madison) according to the manufacturer’s instructions. The HIV-1 pol gene fragment (~1316 bp, encompassing the entire protease region and the first 300 amino acids of reverse transcriptase) was amplified by reverse transcription polymerase chain reaction followed by nested polymerase chain reaction. Amplified products were verified by 1% agarose gel electrophoresis, visualized with deoxyribonucleic acid markers, and purified before bidirectional sequencing (Huahuachen Technology Co., Beijing, China).

### 2.3. Sequence processing and subtype determination

Raw chromatograms were edited and assembled using Sequencher version 5.4.6 (Gene Codes Corp., Ann Arbor). Multiple sequence alignment was performed with BioEdit version 7.2.0 (Ibis Biosciences, Carlsbad), including representative international reference sequences downloaded from the Los Alamos HIV Sequence Database. Phylogenetic analysis was conducted using the Neighbor-Joining method with 1000 bootstrap replicates in Molecular Evolutionary Genetics Analysis version 7.0 (Institute for Genomics and Evolutionary Medicine, Temple University, Philadelphia). Preliminary subtype classification was determined according to the clustering of study sequences with reference strains. Subtypes were further confirmed using the HIV Basic Local Alignment Search Tool to ensure accuracy of classification.

### 2.4. Construction of the molecular transmission network

Pairwise genetic distances among HIV-1 pol sequences were calculated using the TN93 model implemented in HyPhy version 2.2.4. The optimal genetic-distance threshold was determined by evaluating the number of clusters and network connections across a series of thresholds, in line with the Technical Guidelines for HIV Transmission Network Monitoring and Intervention (Trial, 2021) issued by the Chinese Center for Disease Control and Prevention. A threshold of 1.0% genetic distance was selected for network construction. The molecular transmission network was visualized using Cytoscape version 3.6.1. In the network, nodes represented individual HIV-1 sequences (corresponding to patients), and edges indicated genetic linkage between sequences. Clusters were defined as groups containing at least 2 nodes. Nodes with a degree (number of connections) of ≥4 were defined as high-transmission-risk cases.

### 2.5. Statistical analysis

Data were entered into a database created with Microsoft Excel and analyzed using Statistical Package for the Social Sciences version 28.0 (IBM Corp., Armonk). Categorical variables were summarized as frequencies and proportions. Differences between clustered and nonclustered cases were compared using the chi-square test or Fisher’s exact test, as appropriate. A 2-sided *P* < .05 was considered statistically significant.

## 3. Results

### 3.1. Baseline characteristics

From 2022 to 2024, a total of 147 newly diagnosed HIV/AIDS cases were reported in Dinghai District. Whole blood samples were collected from 121 patients, and 117 high-quality pol gene sequences were successfully obtained. Among these patients, 102 were male (87.18%), and 54 were aged ≥50 years (46.15%). With respect to marital status, 55 individuals were married (47.01%). The majority of participants (84 cases, 71.79%) had an educational level of junior high school or below. A total of 74 patients (63.25%) were employed at the time of diagnosis, whereas 52 cases (44.44%) were nonlocal residents, originating from 16 different provinces or municipalities. Most patients were identified through passive testing (98 cases, 83.76%). Regarding transmission routes, 45 cases (38.46%) reported commercial heterosexual contact, 30 cases (25.64%) reported noncommercial heterosexual contact, 32 cases (27.35%) were infected through MSM, and 10 cases (8.55%) were infected by a spouse or steady partner who was HIV-positive.

### 3.2. HIV-1 subtype distribution

**Figure 1.**
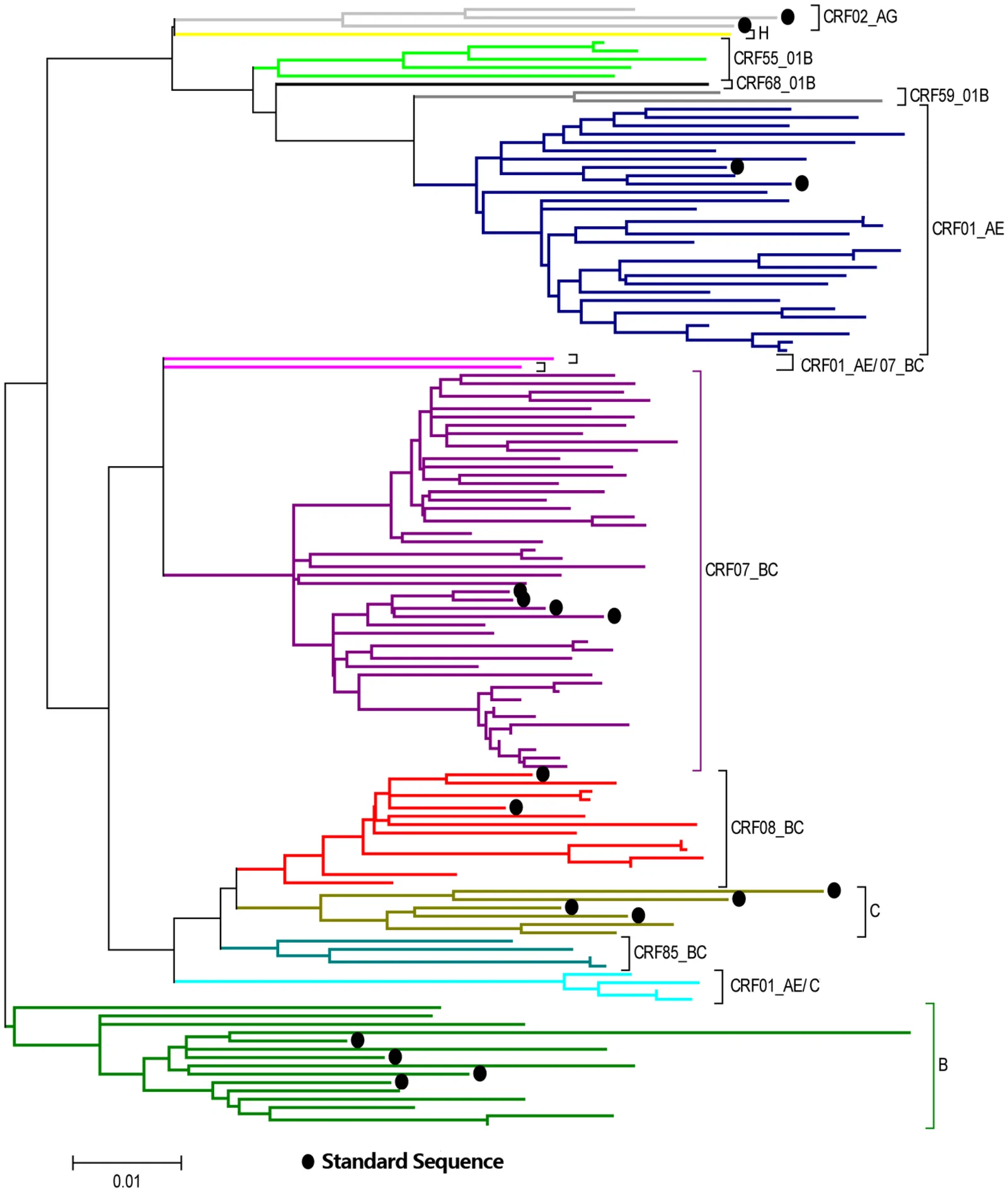
Phylogenetic tree of HIV-1 pol gene sequences from Dinghai District, 2022 to 2024. CRF = circulating recombinant form, HIV-1 = human immunodeficiency virus type 1.

Phylogenetic analysis of the pol gene sequences using the Neighbor-Joining method revealed 13 distinct HIV-1 subtypes and recombinant forms. The predominant subtypes were CRF07_BC (44 cases, 37.61%) and CRF01_AE (28 cases, 23.93%). Other subtypes included CRF08_BC (12 cases, 10.26%), subtype B (11 cases, 9.40%), CRF55_01B (5 cases, 4.27%), and CRF85_BC (4 cases, 3.42%). Less frequently detected forms were CRF59_01B (2 cases, 1.71%), subtype C (2 cases, 1.71%), CRF02_AG (1 case, 0.85%), CRF68_01B (1 case, 0.85%), and subtype H (1 case, 0.85%). In addition, 2 URFs were identified, including URF(CRF01_AE/C; 4 cases, 3.42%) and URF(CRF01_AE/07_BC; 2 cases, 1.71%). In the phylogenetic tree, CRF07_BC, CRF08_BC, and subtype C clustered closely within a major branch, whereas CRF01_AE and subtype B formed distinct, more divergent branches (Fig. 1).

### 3.3. Temporal trends in subtype prevalence

Analysis of subtype distribution over time indicated that the proportion of CRF07_BC and CRF08_BC decreased from 41.86% and 13.95% in 2022 to 35.90% and 7.69% in 2024, respectively. Conversely, the proportion of CRF01_AE increased from 18.60% in 2022 to 31.43% in 2024. Several newly emerging recombinant forms, including CRF55_01B, CRF59_01B, CRF68_01B, and CRF85_BC, were also detected during the study period. However, the differences in subtype distribution between CRF07_BC, CRF01_AE, CRF08_BC, and other subtypes were not statistically significant (χ^2^ = 2.376; *P* > .05).

### 3.4. Clustering characteristics

At the genetic-distance threshold of 1.0%, 14 molecular clusters (C1–C14) were identified, comprising 36 sequences, with an overall clustering rate of 30.77% (36/117). Cluster sizes ranged from 2 to 10 nodes. Patients included in clusters were predominantly male (29/36, 80.56%), aged ≥50 years (27/36, 75.00%), and had an educational level of junior high school or below (32/36, 88.89%). The majority were local residents (30/36, 83.33%) and acquired infection through heterosexual transmission (26/36, 72.22%). The clustered sequences represented 7 different subtypes, with CRF07_BC showing the highest clustering rate (16/36, 44.44%), followed by CRF01_AE (6/36, 16.67%) and CRF08_BC (6/36, 16.67%). Univariate analysis demonstrated that age, marital status, educational level, place of residence, and transmission route were significantly associated with clustering (*P* < .05). Specifically, patients aged ≥50 years, those who were divorced or widowed, those with lower educational attainment, local residents, and individuals infected through spousal or steady partner transmission had higher clustering rates (Table 1).

**Table 1:** Clustering of HIV/AIDS cases in Dinghai District (n [%]).

Variable	Total (n = 117)	Clustered (n = 36)	Nonclustered (n = 81)	χ^2^	*P*
Sex				–	.229
Male	102 (87.18)	29 (28.43)	73 (71.57)		
Female	15 (12.82)	7 (46.67)	8 (53.33)		
Age, yr				18.061	<.001
<30	16 (13.68)	1 (6.25)	15 (93.75)		
30–49	47 (40.17)	8 (17.02)	39 (82.98)		
≥50	54 (46.15)	27 (50.00)	27 (50.00)		
Marital status				11.075	.004
Unmarried	26 (22.22)	2 (7.69)	24 (92.31)		
Married	55 (47.01)	17 (30.91)	38 (69.09)		
Divorced/widowed	36 (30.77)	17 (47.22)	19 (52.78)		
Education level				7.523	.023
Junior high school or below	84 (71.79)	32 (38.10)	52 (61.90)		
High school/technical secondary school	18 (15.38)	2 (11.11)	16 (88.89)		
College or above	15 (12.82)	2 (13.33)	13 (86.67)		
Occupation				1.320	.250
Employed	74 (63.25)	20 (27.03)	54 (72.97)		
Unemployed*	43 (36.75)	16 (37.21)	27 (62.79)		
Residence				16.745	<.001
Local (county)	34 (29.06)	17 (50.00)	17 (50.00)		
Other districts in Zhoushan	31 (26.50)	13 (41.94)	18 (58.06)		
Other provinces/municipalities	52 (44.44)	6 (11.54)	46 (88.46)		
Mode of detection				1.005	.316
Voluntary testing†	19 (16.24)	4 (21.05)	15 (78.95)		
Passive testing‡	98 (83.76)	32 (32.65)	66 (67.35)		
Transmission route				10.071	.018
Commercial heterosexual contact§	45 (38.46)	17 (37.78)	28 (62.22)		
Noncommercial heterosexual contact‖	30 (25.64)	9 (30.00)	21 (70.00)		
MSM	32 (27.35)	4 (12.50)	28 (87.50)		
Spousal/steady partner positive	10 (8.55)	6 (60.00)	4 (40.00)		
Subtype				4.537	.209
CRF07_BC	44 (37.61)	16 (36.36)	28 (63.64)		
CRF01_AE	28 (23.93)	6 (21.43)	22 (78.57)		
CRF08_BC	12 (10.26)	6 (50.00)	6 (50.00)		
Others	33 (28.21)	8 (24.24)	25 (75.76)		

AIDS = acquired immunodeficiency syndrome, CRF = circulating recombinant form, HIV-1 = human immunodeficiency virus type 1, MSM = men who have sex with men.

* Unemployed includes housework, job-seeking, and retired individuals.

† Voluntary testing includes voluntary counseling and testing (VCT) and self-testing.

‡ Passive testing includes health examinations, routine outpatient testing, presurgical screening, and partner notification.

§ Commercial heterosexual contact refers to nonmarital paid heterosexual intercourse.

‖ Noncommercial heterosexual contact refers to nonmarital, unpaid heterosexual intercourse.

### 3.5. Molecular-network analysis

**Figure 2.**
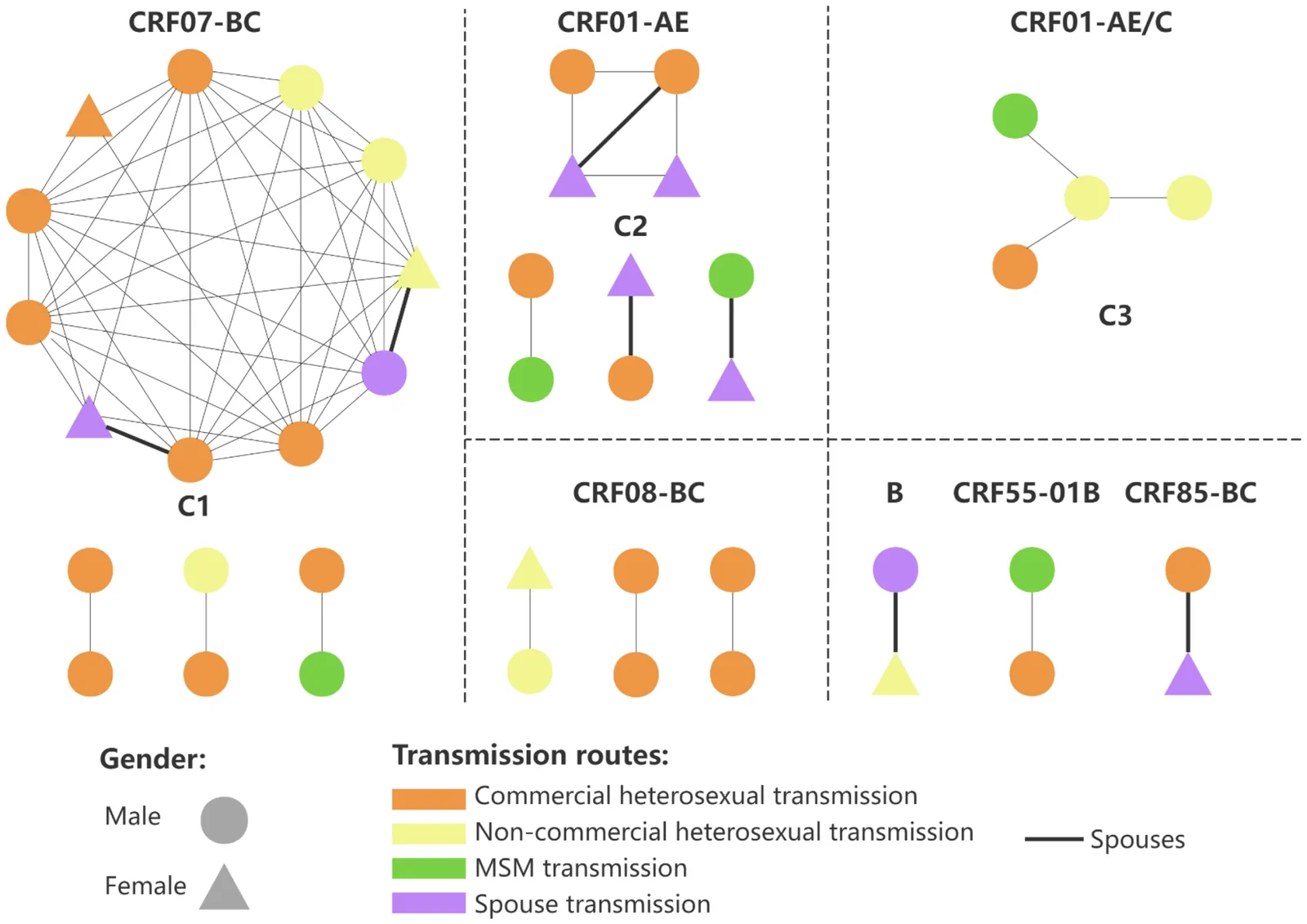
HIV-1 molecular transmission network among newly diagnosed HIV/AIDS cases in Dinghai District (2022–2024). AE = recombinant form comprising HIV-1 subtypes A and E, AIDS = acquired immunodeficiency syndrome, BC = recombinant form comprising HIV-1 subtypes B and C, CRF = circulating recombinant form, HIV-1 = human immunodeficiency virus type 1, MSM = men who have sex with men.

Among the 36 clustered sequences, 7 distinct subtypes were represented. The CRF07_BC subtype formed 4 separate clusters, whereas CRF08_BC and CRF01_AE each formed 3 clusters. Other subtypes were represented by single small clusters. One active transmission cluster (C1) was identified, consisting of 10 CRF07_BC sequences. Temporal analysis showed that this cluster included 5 cases diagnosed in 2022, 3 additional cases in 2023, and 2 cases in 2024. Within this cluster, 9 individuals exhibited high-transmission risk, defined as having ≥4 network edges. These high-risk cases were predominantly aged ≥50 years (7 cases) and male (8 cases). All resided within the district and had an educational level of junior high school or below. Transmission routes in this cluster were mainly heterosexual, with 5 cases reporting commercial heterosexual contact, 3 reporting noncommercial heterosexual contact, and 1 reporting spousal transmission. The C1 cluster was therefore identified as a middle-aged and elderly heterosexual transmission cluster, predominantly involving commercial heterosexual contact. No aggregation of MSM cases was observed. Additionally, clusters C5, C7, C12, and C14 represented spousal transmission networks, while clusters C3, C6, and C13 displayed overlapping transmission involving both MSM and commercial heterosexual contacts (Fig. 2).

## 4. Discussion

This molecular epidemiology study delineated the subtype composition and molecular transmission network of HIV-1 among newly diagnosed HIV/AIDS cases in Dinghai District from 2022 to 2024. Thirteen subtypes and recombinant forms were identified, with CRF07_BC and CRF01_AE constituting the predominant lineages, consistent with national patterns in which CRF07_BC, CRF01_AE, and CRF08_BC account for the majority of circulating strains. The detection of additional CRFs (e.g., CRF55_01B, CRF59_01B, CRF68_01B, and CRF85_BC) and URFs underscores sustained viral diversification and the convergence of multiple transmission sources within this coastal, high-mobility setting. The main contribution of this study is that it provides district-level molecular epidemiological evidence for understanding HIV-1 transmission in Dinghai District. Unlike studies that only describe subtype distribution at a broader provincial or municipal level, this study integrated HIV-1 subtype composition with molecular transmission-network analysis and demographic/behavioral information. This approach allowed us to identify not only the diversity of circulating strains but also the population groups and potential transmission links that may require priority attention in local prevention and control. Therefore, the findings provide more direct evidence for optimizing targeted testing, partner services, and community-based interventions in Dinghai District. Nevertheless, molecular-network links should be interpreted as potential genetic relatedness among viral sequences rather than proof of direct person-to-person transmission.

At a 1.0% TN93 genetic-distance threshold, 30.77% of sequences fell into 14 clusters, a clustering rate lower than reports from parts of Jiangxi Province and prior analyses from Zhoushan. Several contextual factors may contribute, including the smaller population base of Dinghai, limited sample size, and a relatively large proportion of nonlocal residents who may have acquired infection outside the district and had shorter residence duration locally, thereby attenuating local genetic linkages. Notably, clustering was concentrated among men, individuals aged ≥50 years, those with lower educational attainment, local household registration, and heterosexual transmission – particularly commercial heterosexual contact. These features suggest active transmission networks among middle-aged and older populations with constrained risk awareness and prevention behaviors, aligning with earlier observations that lower education and limited HIV knowledge are associated with reduced condom use and delayed testing.^[7,8]^

Network topology highlighted 1 active CRF07_BC cluster (C1) of 10 nodes accumulated over 3 consecutive years, within which 9 individuals exhibited high degree (≥4), indicating elevated potential for onward transmission. Epidemiological review revealed that most high-degree nodes were aged ≥50 years, male, locally resident, and reported heterosexual exposure; 5 involved commercial heterosexual contact and 3 noncommercial heterosexual contact, with 1 spousal transmission.^[4,9–11]^ However, this finding should not be interpreted as evidence that this group is the only source or definite “core” of transmission. Molecular clustering reflects genetic similarity among viral sequences under a predefined genetic-distance threshold and cannot determine direct transmission, transmission direction, or the exact time and place of infection. Therefore, these results should be understood as evidence of potential transmission links and used to guide priority prevention and follow-up, rather than as definitive proof of individual-level transmission relationships.

The dominance of CRF07_BC and CRF01_AE, together with the presence of multiple recombinant forms, indicates a heterogeneous epidemic that requires stratified prevention. Evidence from the network suggests that intensified interventions should prioritize men aged ≥50 years with lower educational attainment; patrons and workers at commercial sex venues and adjacent informal settings; and spouses/steady partners of persons with newly diagnosed HIV. Operationally, this translates to expanding community-based testing in older age groups, scaling venue- and neighborhood-focused outreach in identified hotspots, reinforcing condom promotion, and ensuring rapid linkage to care and partner services. For spousal transmission, standardized partner notification with facilitated testing and same-day antiretroviral therapy (ART) initiation may reduce within-household spread.^[12,13]^

The overall subtype distribution aligns with national surveillance indicating CRF07_BC and CRF01_AE as leading lineages, whereas the detection of CRF08_BC and multiple newer CRFs mirrors evolving recombinant dynamics reported in other Chinese regions. The lower clustering rate relative to selected provinces and prior local data is plausible given demographic flux and sampling frames; nevertheless, the identification of an active CRF07_BC cluster accruing new cases across years indicates sustained local transmission that merits focused disruption.^[14]^

The public health implications of these findings are mainly reflected in targeted prevention and early interruption of potential transmission chains. First, health education should be strengthened among priority populations identified by the molecular network, especially regarding condom use, HIV testing after risk exposure, and awareness of early ART. Second, community-based testing, provider-initiated testing, and outreach testing should be expanded to improve early case detection. Third, partner notification and assisted partner services should be promoted under voluntary, confidential, and nondiscriminatory principles, so that potential contacts can receive timely counseling and testing. Fourth, newly diagnosed individuals should be linked rapidly to ART, viral-load monitoring, and adherence support. Finally, molecular-cluster information may be used as an auxiliary tool for public health follow-up, helping local health departments prioritize health education, testing resources, and community interventions for groups or networks with higher clustering signals.

This analysis integrates phylogenetic subtyping with network inference using a standardized pol fragment and a prespecified genetic distance threshold. Several limitations should be acknowledged when interpreting the results. First, this study was conducted in a single district, and the findings mainly reflect the molecular epidemiological characteristics of HIV-1 in Dinghai District during the study period; therefore, generalization to other regions should be made with caution. Second, only samples with successful sequence amplification and available epidemiological information were included, which may have introduced selection bias. Third, the molecular transmission network was constructed using partial HIV-1 pol sequences and a predefined genetic-distance threshold; different genomic regions or thresholds may lead to different network structures. Fourth, molecular-network analysis can identify genetic relatedness among viral sequences but cannot prove direct transmission, infer transmission direction, or determine the exact time and place of infection. Fifth, behavioral information such as sexual contact history may be affected by recall bias, social desirability bias, or underreporting. Finally, because this was an observational analysis based on available surveillance data, the dynamic evolution of transmission clusters could not be fully evaluated. Future studies should combine longitudinal sequence surveillance, more detailed epidemiological investigation, viral-load data, and multicenter comparisons to further validate these findings.

## 5. Conclusions

In conclusion, this study characterized the HIV-1 subtype distribution and molecular transmission-network features in Dinghai District and identified population groups with relatively higher clustering signals. The findings suggest that older individuals may play an important role in local transmission and should be considered a priority population for targeted prevention, early testing, partner services, and timely ART. These results provide district-level evidence for improving local HIV prevention and control strategies. However, because molecular-network links cannot confirm direct transmission or transmission direction, the conclusions should be interpreted cautiously and validated through continued molecular surveillance and epidemiological follow-up.

## Author contributions

**Conceptualization:** Youshu Wang, Bo Zhang, Songye Gu, Qizhen Yu, Junwei Chen.

**Data curation:** Youshu Wang, Bo Zhang, Songye Gu, Qizhen Yu, Junwei Chen.

**Formal analysis:** Youshu Wang, Bo Zhang, Songye Gu, Qizhen Yu, Junwei Chen.

**Funding acquisition:** Junwei Chen.

**Investigation:** Junwei Chen.

**Writing – original draft:** Junwei Chen.

**Writing – review & editing:** Junwei Chen.
